# Micro-Raman Spectroscopy and X-ray Diffraction Analyses of the Core and Shell Compartments of an Iron-Rich Fulgurite

**DOI:** 10.3390/molecules27103053

**Published:** 2022-05-10

**Authors:** Ahmet Karadag, Ersin Kaygisiz, Timur Nikitin, Sinan Ongen, Gulce Ogruc Ildiz, Namik Aysal, Ayberk Yilmaz, Rui Fausto

**Affiliations:** 1 Department of Physics, Faculty of Sciences and Letters, Istanbul Kultur University, Istanbul 34158, Turkey; karadagahmet23@gmail.com; 2 Department of Geological Engineering, Faculty of Engineering, Istanbul University-Cerrahpasa, Istanbul 34320, Turkey; ersinkygsz@gmail.com (E.K.); asinanongen@gmail.com (S.O.); aysal@iuc.edu.tr (N.A.); 3 Department of Chemistry, University of Coimbra, CQC-IMS, 3004-535 Coimbra, Portugal; timur.nikitin@uc.pt (T.N.); rfausto@ci.uc.pt (R.F.); 4 Department of Physics, Faculty of Science, Istanbul University, Istanbul 34134, Turkey; ayberk@istanbul.edu.tr

**Keywords:** mineralogical composition, iron-rich fulgurite, Raman spectroscopy, X-ray diffraction, infrared spectroscopy, chemometrics, SEM-BSE, SEM-EDX, polarized optical microscopy

## Abstract

Fulgurites are naturally occurring structures that are formed when lightning discharges reach the ground. In this investigation, the mineralogical compositions of core and shell compartments of a rare, iron-rich fulgurite from the Mongolian Gobi Desert were investigated by X-ray diffraction and micro-Raman spectroscopy. The interpretation of the Raman data was helped by chemometric analysis, using both multivariate curve resolution (MCR) and principal component analysis (PCA), which allowed for the fast identification of the minerals present in each region of the fulgurite. In the core of the fulgurite, quartz, microcline, albite, hematite, and barite were first identified based on the Raman spectroscopy and chemometrics analyses. In contrast, in the shell compartment of the fulgurite, the detected minerals were quartz, a mixture of the K-feldspars orthoclase and microcline, albite, hematite, and goethite. The Raman spectroscopy results were confirmed by X-ray diffraction analysis of powdered samples of the two fulgurite regions, and are consistent with infrared spectroscopy data, being also in agreement with the petrographic analysis of the fulgurite, including scanning electron microscopy with backscattering electrons (SEM-BSE) and scanning electron microscopy with energy dispersive X-ray (SEM-EDX) data. The observed differences in the mineralogical composition of the core and shell regions of the studied fulgurite can be explained by taking into account the effects of both the diffusion of the melted material to the periphery of the fulgurite following the lightning and the faster cooling at the external shell region, together with the differential properties of the various minerals. The heavier materials diffused slower, leading to the concentration in the core of the fulgurite of the iron and barium containing minerals, hematite, and barite. They first underwent subsequent partial transformation into goethite due to meteoric water within the shell of the fulgurite. The faster cooling of the shell region kinetically trapped orthoclase, while the slower cooling in the core area allowed for the extensive formation of microcline, a lower temperature polymorph of orthoclase, thus justifying the prevalence of microcline in the core and a mixture of the two polymorphs in the shell. The total amount of the K-feldspars decreases only slightly in the shell, while quartz and albite appeared in somewhat larger amounts in this compartment of the fulgurite. On the other hand, at the surface of the fulgurite, barite could not be stabilized due to sulfate lost (in the form of SO_2_ plus O_2_ gaseous products). The conjugation of the performed Raman spectroscopy experiments with the chemometrics analysis (PCA and, in particular, MCR analyses) was shown to allow for the fast identification of the minerals present in the two compartments (shell and core) of the sample. This way, the XRD experiments could be done while knowing in advance the minerals that were present in the samples, strongly facilitating the data analysis, which for compositionally complex samples, such as that studied in the present investigation, would have been very much challenging, if possible.

## 1. Introduction

Fulgurites are natural structures formed by the rapid heating of rock, sand, or soil by a cloud-to-ground lightning strike, and they take their name from the Latin term for lightning, *fulgur* [1]. Lightnings are natural phenomena that occur at a global flash rate of about 45–65 times per second on Earth [2], mostly (75–90%) by transmission of electricity from the cloud to the continental land mass. Theoretically, fulgurites are natural structures with a high compositional diversity as they can be formed from anything struck by lightning. They are divided into five classes, as sand fulgurites, clay fulgurites, caliche fulgurites, rock fulgurites, and droplet fulgurites, according to their morphology [3].

As it is difficult to directly observe the properties of lightning passing through the soil, including the energy deposited (between 1 and 30 MJ m^–1^), lightning channel width (~1 mm diameter), and peak temperatures (in the order of 1000 K s^–1^), fulgurites are potentially a source of data to investigate the heating and cooling rates associated with lightning effects on the ground, providing important clues about the properties of lightning moving underground [4]. The differential mineral compositions within the fulgurites, in particular those observed from the inner part of the fulgurites (core compartment) and the more external domain (shell), are also a relevant piece of information and, because of that, the mineral composition of fulgurites and their change in going from core to shell regions have been the subject of several studies, aiming at establishing different types of correlations between these composition or/and compositional changes, as well as the fulgurites’ formation mechanism [2,3,4,5,6,7,8,9].

In this study, an iron-rich fulgurite sample from the Gobi Desert in Mongolia has been investigated, this study being, to the best of our knowledge, the first investigation dealing with a fulgurite from this geographic location reported hitherto. The mineral contents of the core and shell compartments of the fulgurite samples were evaluated by using a combined Raman micro-spectroscopy, X-ray powder diffraction, scanning electron microscopy with backscattering electrons (SEM-BSE) and scanning electron microscopy with energy-dispersive X-ray (SEM-EDX) approach. The Raman data were used for fast non-destructive identification of the minerals present in the fulgurite, and the data analysis was performed with help of chemometrics (principal component analysis—PCA—and multivariate curve resolution—MCR—methods). The conjugation of the performed Raman spectroscopy experiments with the chemometrics analysis, in particular, MCR analyses) was shown to allow the fast identification of the minerals present in the two compartments (shell and core) of the sample. This way, the XRD experiments could be done while knowing in advance the minerals present in the samples, this way strongly facilitating the data analysis, which, for compositionally complex samples such as that studied in the present investigation, would have been very much challenging, if possible.

As it will be described in detail in the next sections of this article, the mineral composition of the core and shell compartments of the studied fulgurite are distinct, with quartz, microcline, albite, hematite, and barite existing in the core, and quartz, a mixture of K-feldspars (orthoclase and microcline), albite, hematite, and goethite being present in the shell region. The different minerals were promptly identified by Raman spectroscopy coupled to chemometrics, and their relative abundance was estimated semi-quantitatively from the powder XRD data, the conclusions of these analysis being also in agreement with the petrographic analysis of the fulgurite and SEM-BSE and SEM-EDX data. The differences in the mineral composition were explained, taking into account the differential diffusion rates at the time of the lightning, the different cooling rates at surface and in the interior of the fulgurite after lightning, and the specific characteristics of the minerals.

## 2. Materials and Methods

The fulgurite sample that was investigated in this study was cut from a fulgurite found in the Mongolian Gobi Desert, West of the city of Dalanzadgad (Tugrugiin Shire). The fulgurite is an iron-rich fulgurite, exhibiting the characteristic cylindrical shape and a high-crystallinity degree [3,10]. Detailed petrographic observations of the fulgurite indicate that it is derived from the desert sands. The fact that the mineral grains in the fulgurite are mostly angular in shape indicates that they were formed as a result of physical disintegration with the effect of heating–cooling in place. Thin sections (~1 mm thickness) of the provided sample were obtained and used in the analyses.

**Figure 1:**
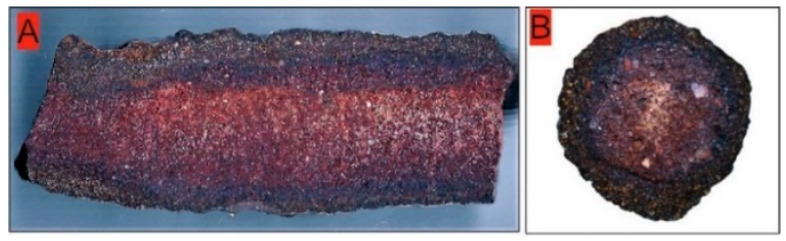
Cut side view (**A**) and upright view (**B**) of the studied fulgurite.

The thin sections exhibit a distinctive color and texture differences in the outer shell and inner (core) compartments (Figure 1). The sections were examined under the microscope (Leitz Orthoplan Widefield Trinocular Smith-T DIC Research Microscope ICT Fluotar) under polarized light, revealing presence of multiple crystalline materials in both the shell and core parts of the sample, with the amount of glassy material (SiO_2_; lechatelierite) being very minor (<3%).

The Raman spectra were obtained using either a portable B&W-Tek i-Raman Plus-785 model or a Horiba LabRam HR Evolution micro-Raman system, equipped with a 785 nm excitation laser set to provide a power of 40 mW at the sample. For minerals’ identification based on the Raman spectra, a chemometrics-based strategy was designed that used both multivariate curve resolution (MCR) [11] and principal component analysis (PCA) [12], as described below. In the measurements made using the B&W-Tek instrument, the spectra were collected with an integration time of 30 s, with 32 scans being accumulated and averaged. In the measurements made using the Horiba spectrometer, the integration time and number of accumulations were 10 s and 10, respectively. In both cases, a 50× objective (numerical aperture: 0.55) was used (spot size, 1.7 μm), and the spectra were collected with a resolution of 4 cm^–1^. The data analysis for minerals identification was done within the 200–1100 cm^−1^ range. Calibration of the spectrometers was done using the characteristic Si wafer band at 520.5 cm^−1^. The accuracy in the measured wavenumbers is better than 0.5 cm^−1^.

Attenuated total reflectance (ATR) infrared spectra (1 cm^−1^ resolution) were recorded in the 450–1750 cm^−1^ wavenumber range using a diamond GladiATR accessory (Pike Technologies) on a Perkin Elmer Spectrum One Fourier transform infrared (FTIR) spectrometer, equipped with a Ge/KBr beam splitter and a deuterated triglycine sulfate (DTGS) detector. Sixty-four scans were co-added to produce each spectrum.

X-ray powder diffraction patterns were collected at room temperature using a GNR APD-2000 Pro diffractometer at 2*θ* = 1°/min goniometer speed and CuKα radiation (λ = 1.5418 Å; 40 kV; 30 mA). A Cu anode was used for the analysis, but although Fe-containing minerals were detected, no fluorescence effect was observed in the X-ray pattern. For data collection, a 2*θ* scan range between 5 and 55° was used, with a step size of 0.02°, a divergence slit of 0.5 mm, and a receiving slit of 0.3 mm. Fractions of the shell and core compartments of the fulgurite were gently removed with a razor blade, and the resulting powder was grinded with an agate mortar and pestle to 150 mesh size (~90 μm). Mineralogical analysis and semi-quantitative determination of minerals present in the samples were done with the program Match! [13], using the reference intensity ratio method (RIR) [14] with mineral reference diffraction data taken from the Crystallography Open Database (COD) [15,16,17,18,19].

Grain–matrix ratios of the fulgurite samples were calculated from petrographic thin sections using image analysis software. For this purpose, overlapping microphotographs were taken on the sections and then, these photos were combined panoramically. A Leitz Orthoplan microscope, the Leica image analysis system, and the Leica Application Suite software were used for these processes. The panoramically combined photographs were determined over the areas covered by the grains and the matrix using a series of graphic software (Adobe Photoshop and Autocad).

The scanning electron microscope–energy-dispersive X-ray spectroscopy (SEM-EDX) analyses were performed by using a Hitachi SU3500 T2 SEM device and an attached energy dispersive X-ray (EDX) spectrometer (Oxford XACT) in the Fatih Sultan Mehmet Vakıf University Aluminum Testing, Education, and Research Center (ALUTEAM). The analyses were carried out using an accelerating voltage of 15 kV, a beam current of 100 nA, and a beam size of 0.6 µm.

Chemometric analyses of the Raman data were performed using the Unscrambler^TM^ software (Version 10.5) [20]. The spectra were pre-processed by performing baseline correction using the OMNIC (Version 8.2.0.387) software [21], followed by median filter 5-points smoothing and vector normalization using Unscrambler^TM^. Principal component analysis (PCA) was performed by using the singular value decomposition (SVD) algorithm [12] and cross validation (random with 20 segments). Multivariate curve resolution (MCR) used the alternating least squares algorithm and was applied without initial guess, under non-negativity (on both concentration and spectra) and closure constraints [11]. The total number of samples (Raman spectra, 200–1100 cm^−1^ range) used in each PCA and MCR calculation was 60.

## 3. Results and Discussion

### 3.1. Raman Spectroscopy and Chemometrics Analyses

**Figure 2:**
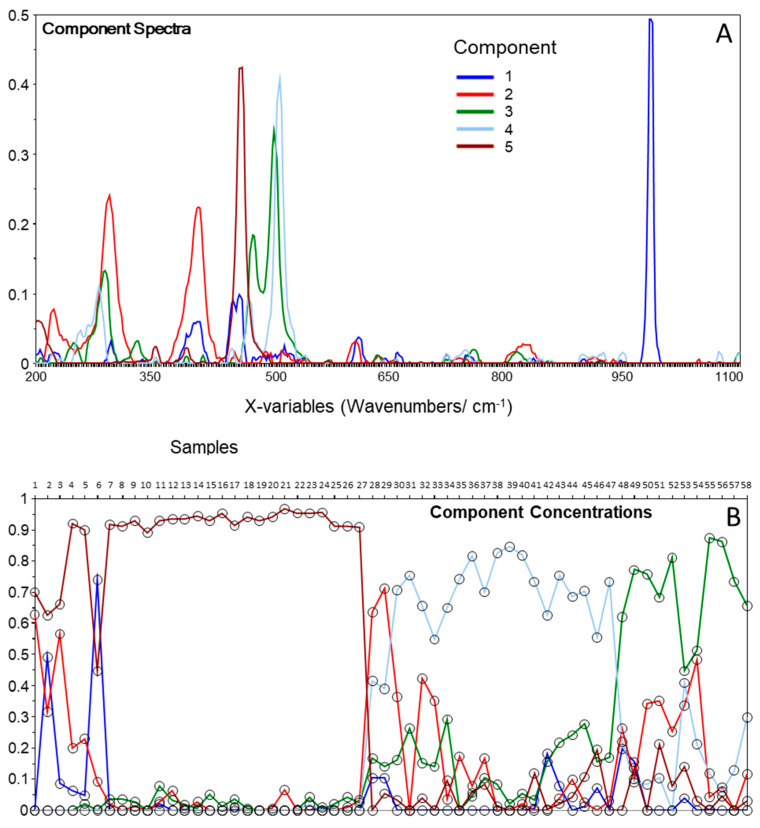
Components’ spectra (**A**) and components’ concentrations (**B**) plots resulting from MCR analysis of the Raman spectra of the core section of the fulgurite. The components’ spectra were vector-normalized.

Sixty Raman spectra were collected at randomly located spots of the core and shell compartments of the fulgurite that were identified as crystalline materials under polarized light, in a total of 120 spectra. The spectra were processed as described in Section 2, and two spectra of the core region were eliminated from the dataset during the chemometrics analysis, due to an insufficient signal-to-noise ratio. MCR was then applied to each set of spectra (core and shell). The components’ spectra and components’ concentration plots for the core of the fulgurite are shown in Figure 2.

**Figure 3:**
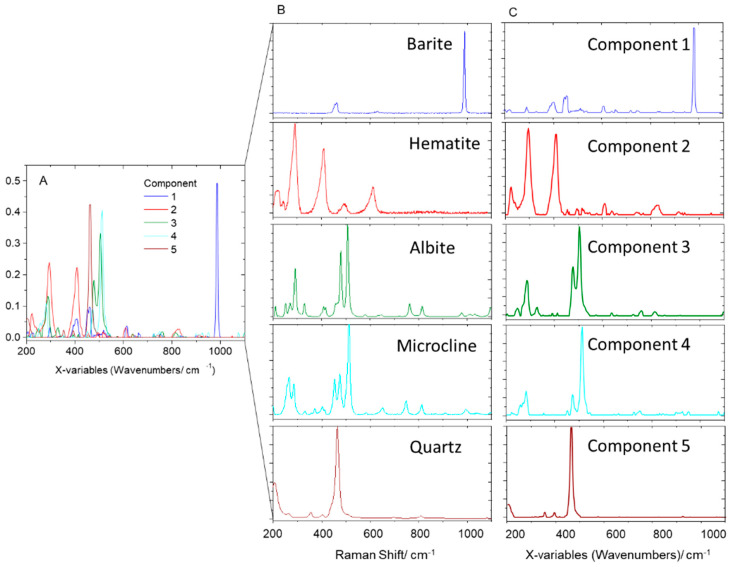
MCR components’ spectra (**A**,**C**) and Raman spectra of the identified minerals in the core section of the studied fulgurite (**B**). The components’ spectra were vector-normalized in A and normalized by the most intense peak in C; minerals’ spectra were normalized by the most intense peak. RRUFF reference spectra: barite, X050029.4; hematite, R060190; albite, X050005; microcline, R120005; quartz, R110108.

For the core of the fulgurite, the MCR decomposition yielded five components (Figure 2A), which allowed us to organize the samples into three main classes, with dominant contributions from components three, four, and five, each one exhibiting sub-classes related to contributions from components one and two (Figure 2B). The comparison of the components’ spectra with reference Raman spectra of minerals in the RRUFF database [22] allowed us to promptly assign components one to five to barite (BaSO_4_), hematite (Fe_2_O_3_), the plagioclase feldspar albite (NaAlSi_3_O_8_), the K-feldspar microcline (KAlSi_3_O_8_), and quartz (SiO_2_), respectively (Figure 3).

**Figure 4:**
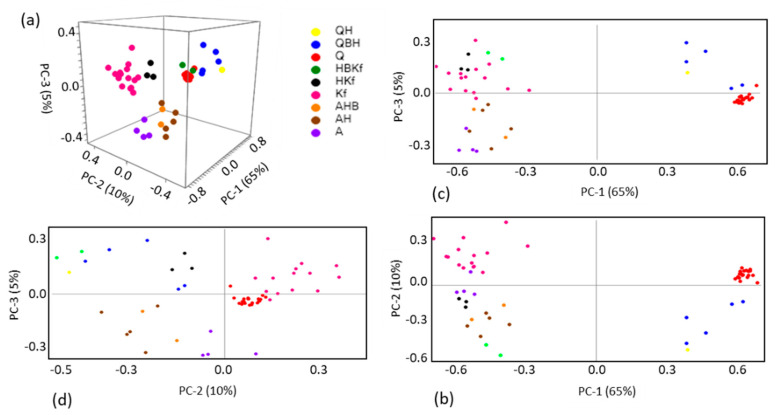
PCA scores plots ((**a**) PC-3 *vs.* PC-2 *vs.* PC-1, (**b**) PC-2 *vs.* PC-1, (**c**) PC-3 *vs.* PC-1, (**d**) PC-3 *vs.* PC-2) resulting from the analysis of the Raman spectra of the core section of the studied fulgurite. Q: quartz; B: barite; H: hematite; A: albite; Kf: K-fedspar (microcline).

**Figure 5:**
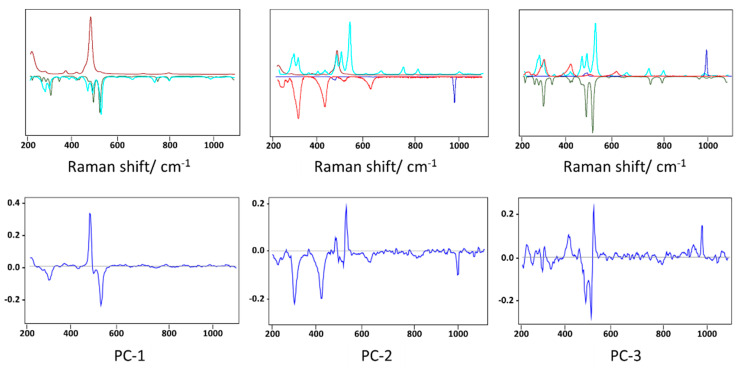
PCA loadings resulting from the analysis of the Raman spectra of the core section of the studied fulgurite (**bottom**) as compared with the Raman spectra of the relevant minerals (**top**). The colors of the spectra of the minerals are the same as those shown in Figure 3.

The PCA score plots for the core compartment of the fulgurite allowed clear identification of the groups of spectra with major contributions of quartz, and the two types of feldspars (albite and microcline) along PC-1, the first with positive scores and the latter with negative scores (Figure 4a–c; score plots of PC-1 vs. PC-2 and PC-3, and a 3D score plot of PC-1 vs. PC-2 vs. PC-3). Along PC-2, pure quartz and microcline samples have positive scores, pure albite samples possess scores around the origin of the axis, and the spectra of the samples containing one of these minerals, plus either hematite alone or hematite and barite simultaneously, have negative scores (Figure 4a,b,d; score plots of PC-2 vs. PC-1 and PC-3, and a 3D score plot of PC-1 vs. PC-2 vs. PC-3). PC-3 well-separates the samples containing microcline (with positive scores) from those containing albite (with negative scores), as shown in Figure 4a,c,d (score plots of PC-3 vs. PC-1 and PC-2 and a 3D scores plot of PC-1 vs. PC2 vs. PC-3). The positions of the different types of spectra in the scores plots can be easily rationalized by looking to the PC-1, PC-2, and PC-3 loadings, and to the corresponding spectra of the minerals, as shown in Figure 5.

**Figure 6:**
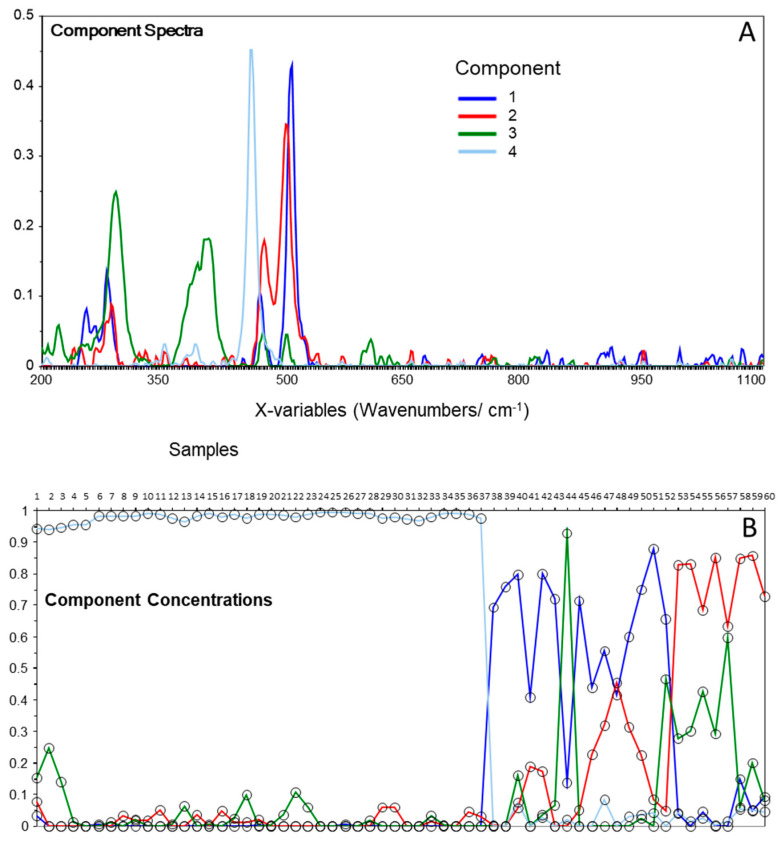
Components’ spectra (**A**) and components’ concentrations (**B**) plots resulting from MCR analysis of the Raman spectra of the shell section of the fulgurite. The components’ spectra were vector-normalized.

**Figure 7:**
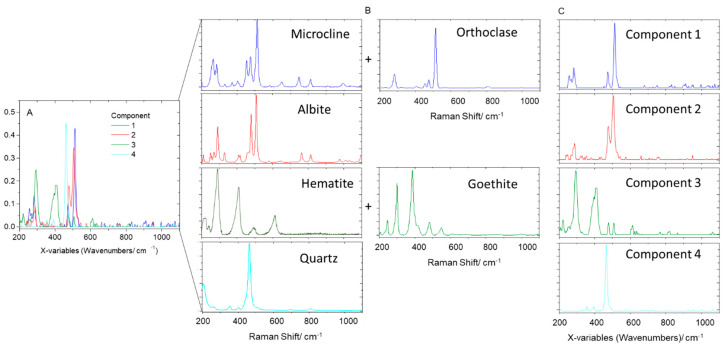
MCR components’ spectra (**A**,**C**) and Raman spectra of the identified minerals in the shell section of the studied fulgurite (**B**). The components’ spectra were vector-normalized in (**A**) and normalized by the most intense peak in (**C**); minerals’ spectra were normalized by the most intense peak. RRUFF reference spectra: microcline, R120005; orthoclase, X050126; albite, X050005; hematite, R060190; goethite, R120086; quartz, R110108.

For the shell section of the sample, the MCR decomposition identified four components (Figure 6a), which again allowed us to classify the samples into three main groups, with dominant contributions from components one, two, and four, while component three is the major constituent in one spectrum and has relevant contributions for a few additional spectra dominated by the other components (Figure 6b). The components two and four were easily identified as being albite and quartz (Figure 7), while components three and one are better described as mixtures of hematite and goethite (FeO(OH)) and of microcline and orthoclase (this latter mineral being a higher-temperature polymorph of microcline), respectively (Figure 7).

**Figure 8:**
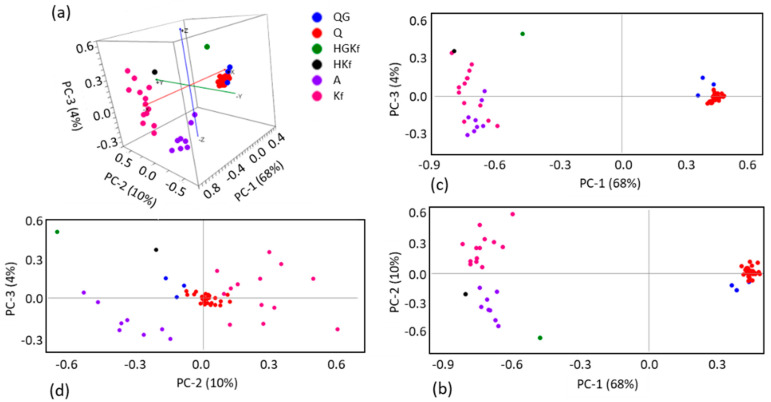
PCA score plots ((**a**) PC-3 *vs.* PC-2 *vs.* PC-1, (**b**) PC-2 *vs.* PC-1, (**c**) PC-3 *vs.* PC-1, (**d**) PC-3 *vs.* PC-2) resulting from the analysis of the Raman spectra of the shell section of the studied fulgurite. Q: quartz; G: goethite; H: hematite; A: albite; Kf: K-fedspars (microcline and orthoclase).

**Figure 9:**
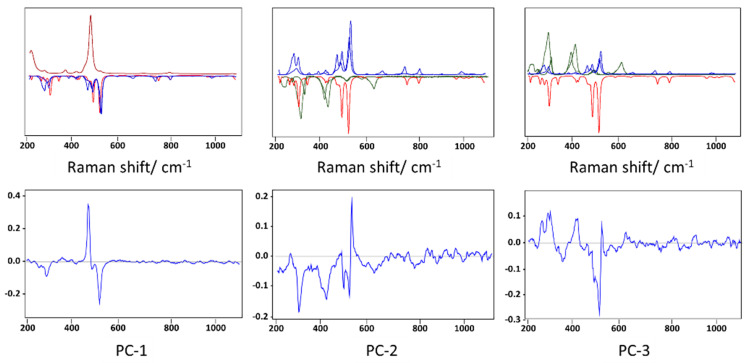
PCA loadings resulting from the analysis of the Raman spectra of the shell section of the studied fulgurite (**bottom**) as compared with the Raman spectra of the relevant minerals (**top**). The colors of the spectra of the minerals are the same as those shown in Figure 7.

The shell PCA scores plots clearly reveal separation of the spectra with major contributions of quartz and the two types of feldspars (plagioclase albite and the K-feldspars microcline and orthoclase) along PC-1, the first with positive scores and the latter with negative scores (Figure 8a–c; score plots of PC-1 vs. PC-2 and PC-3 and a 3D scores plot of PC-1 vs. PC-2 vs. PC-3). Along PC-2, pure quartz samples possess scores around the origin of the axis (mostly in the positive side), while albite samples have negative scores, and the K-feldspars samples have positive ones. Presence of hematite or goethite in the samples led to negative PC-2 scores (Figure 8a,b,d; score plots of PC-2 vs. PC-1 and PC-3, and a 3D scores plot of PC-1 vs. PC2 vs. PC-3). Along PC-3 (Figure 9; score plots of PC-3 vs. PC-1 and PC-2 and a 3D scores plot of PC-1 vs. PC-2 vs. PC-3), the samples with hematite/goethite have positive scores, while most of those containing albite have negative scores. Along PC-3, quartz samples are placed near the origin, and the K-feldspars samples are scattered (mostly in the positive side). Interestingly, the PC-2 vs. PC-3 scores plot shows a very good separation between the different types of samples. Once again, the positions of the different types of spectra in the scores plots can be easily rationalized by looking to the PC-1, PC-2, and PC-3 loadings (see Figure 9) and by comparing these with the spectra of the minerals, shown in Figure 7.

In summary, the Raman data and their chemometric analysis allowed for the fast identification of quartz, microcline, hematite, albite, and barite in the core of the studied fulgurite. Considering the number of occurrences of the minerals in the different spectra, quartz appears as the major component existing in the core compartment of the fulgurite, followed by microcline and albite, with hematite and barite being present in smaller, but still significant amounts. In contrast, in the shell compartment of the fulgurite, the detected minerals were quartz (major component), a mixture of the K-feldspars orthoclase and microcline, the plagioclase feldspar albite, and smaller amounts of hematite and goethite. As shown in Section 3.3, these results are fully supported by the powder XRD results, which were performed on the core and shell samples of the fulgurite.

Assignments for the Raman spectra of the minerals that were identified in the studied fulgurite are given in the Supplementary Material (Figures S1–S7), according to the literature [23,24,25,26,27,28,29,30,31,32,33,34,35].

### 3.2. Infrared Spectroscopy

**Figure 10:**
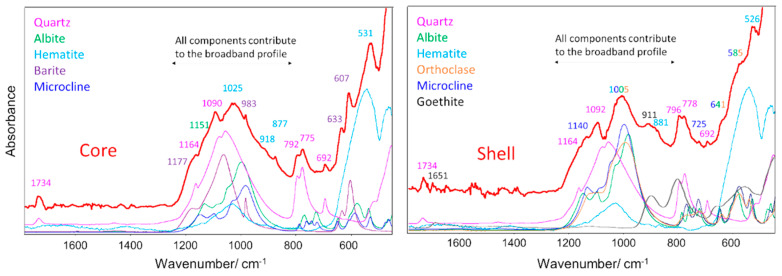
Assignment of the IR (ATR) spectra of the core (**left**) and shell (**right**) compartments of the studied fulgurite (red spectrum). In the region around 900–1200 cm^−1^, glassy SiO_2_ material also contributes to the intensity [36].

The infrared (ATR—attenuated total reflectance) spectra of powdered samples of the material extracted from the two compartments of the fulgurite are shown in Figure 10, together with the spectra of the identified minerals, which were extracted from the RRUFF database [22].

In the Figure, the spectra of the two regions of the fulgurite are shown in red, while the reference spectra of the constituting minerals [22] are given in different colors, which are identified in the legend. The wavenumbers of the maxima of the observed bands in the spectrum of the fulgurite are labeled with the color corresponding to the mineral to which they are assigned (major contributor). In the cases when more than one mineral is considered to contribute extensively to the band, the wavenumbers are written using the different corresponding colors.

The data shown in the Figure clearly demonstrate that the infrared spectra of the fulgurite samples confirm the presence of the minerals that were identified by Raman spectroscopy in each one of the compartments of the fulgurite.

### 3.3. X-ray Powder Diffraction Analysis

**Figure 11:**
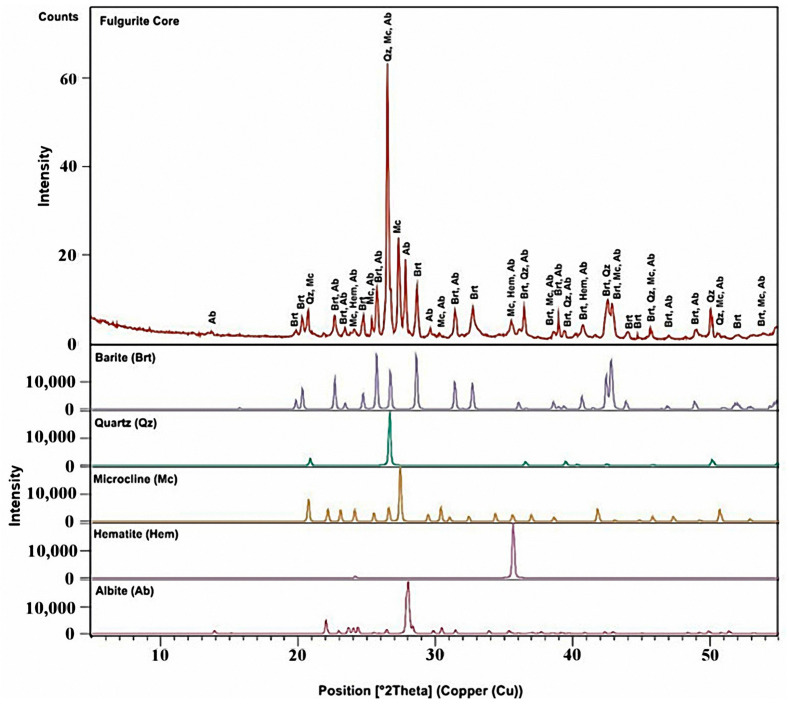
X-ray powder diffraction pattern of the fulgurite core sample. Brt: barite; Qz: quartz; Mc: microcline; Hm: hematite; Ab: albite. The observed diffractogram of the sample corresponds to the red line shown in the top panel of the figure, while the separate contributions of the different minerals present in the sample are shown in different colors in the remaining panels. COD IDs of the reference diffractograms (ICSD Database): quartz, 01-083-0539; barite, 01-089-3749; microcline, 00-019-0926; hematite, 01-072-0469; albite, 00-001-0739.

**Figure 12:**
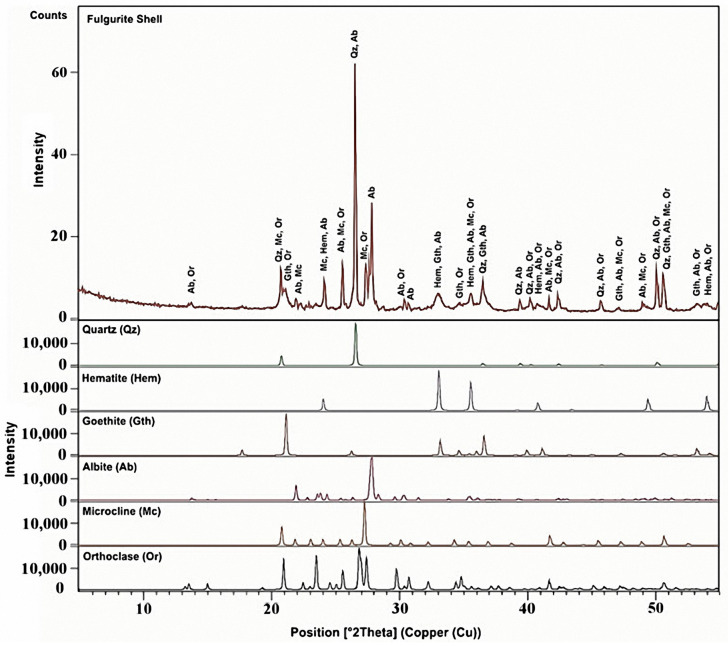
X-ray powder diffraction pattern of the fulgurite core sample. Qz: quartz; Hm: hematite; Gth: goethite; Ab: albite; Mc: microcline; Or: orthoclase. The observed diffractogram of the sample corresponds to the red line shown in the top panel of the figure, while the separate contributions of the different minerals present in the sample are shown in different colors in the remaining panels. COD IDs of the reference diffractograms (ICSD Database): quartz, 01-083-0539; microcline, 00-019-0926; orthoclase, 01-086-0437; hematite, 01-072-0469; albite, 00-001-0739; goethite, 00-017-0536.

The XRD patterns of the powdered material obtained from the fulgurite core and shell compartments are displayed in Figure 11 and Figure 12, respectively. The indexation was found to be fully consistent with the Raman and IR-ATR data presented in Section 3.1 and Section 3.2, testifying to the presence of quartz, microcline, barite, hematite, and albite phases in the core region, and quartz, microcline, orthoclase, albite, hematite, and goethite in the shell. Semi-quantitative calculation of the relative percentages of the different minerals in the samples was performed using the RIR method [14]. For the estimations, the most intense peak of each mineral was used (quartz: 2*θ* = 26.639°, d = 3.34363 Å [37]; microcline: 2*θ* = 27.421°, d = 3.25000 Å [38]; albite: 2*θ* = 28.022°, d = 3.18167 Å [39]; hematite: 2*θ* = 35.640°, d = 2.51710 Å [40]; barite: 2*θ* = 25.856°, d = 3.44305 Å [41]; orthoclase: 2*θ* = 26.914°, d = 3.31000 Å [42]; goethite: 2*θ* = 21.223°, d = 4.18300 Å [43]). The resulting mineral compositions (together with some additional information regarding the minerals) are shown in Table 1.

The percentage amounts of minerals in the core were estimated as being: quartz 52%, microcline 22%, albite 10%, barite 8%, and hematite 8%, while in the shell the obtained values were: quartz 56%, (microcline + orthoclase) (9 + 11) = 20%, albite 15%, hematite 4% and goethite 5%, which are in qualitative agreement with the indications provided by the Raman experiments.

### 3.4. Polarized Optical Microscopy, SEM-BES, and SEM-EDX Studies

**Figure 13:**
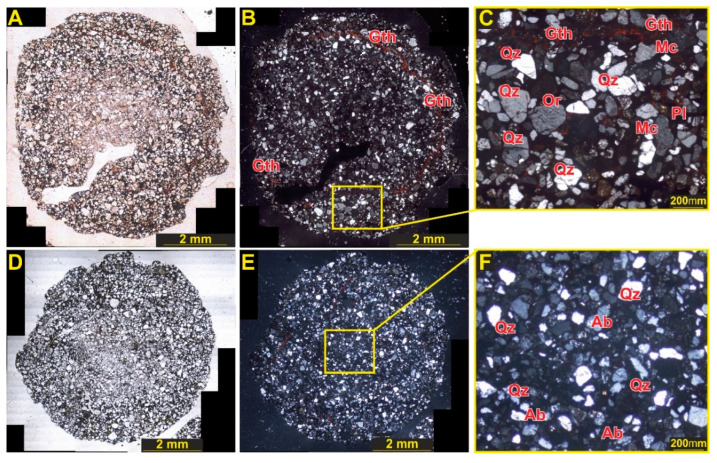
Plain polarized (**A**,**D**), and crossed polarized light (**B**,**E**) views for two different fulgurite sections. Quartz, orthoclase, microcline, and plagioclase in fulgurite and Fe oxides in the matrix for the shell (**C**) and core (**F**). Abbreviations: Qz: quartz, Ab: albite, Or: orthoclase, Mc: microcline, Gth: goethite.

The fulgurite was also subjected to polarized optical microscopy, SEM-BES, and SEM-EDX analyses, which fully confirm the conclusions from the Raman and IR spectroscopies, as well as the XRD studies. Figure 13 shows images of the fulgurite obtained with plain polarized (A,D) and crossed polarized light (B,E). It can be seen from this Figure that quartz, albite, and microcline were observed both in the shell and in the core, while orthoclase was observed only in the shell, in concordance with the results presented in Section 3.1, Section 3.2 and Section 3.3. Similarly, although hematite, one of the Fe oxide minerals present in the fulgurite, was found in both the shell and the core, goethite was observed only in the shell. 

**Figure 14:**
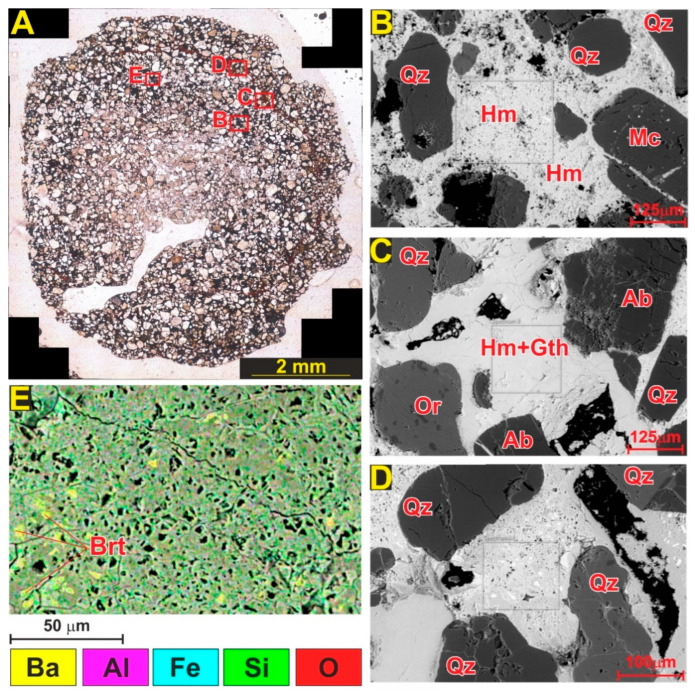
BSE images and elemental mapping of selected areas in core and shell section of fulgurite samples. (**A**) SEM-BSE and SEM-EDX analysis location on fulgurite sample. SEM-BSE images of the core (**B**), and shell sections (**C**,**D**). SEM-EDX mapping on the core section (**E**) of fulgurite. Abbreviations: Qz: quartz, Ab: albite, Mc: microcline, Or: orthoclase, Brt: barite.

In the petrographic examinations, no barite mineral was observed in thin section scale. However, as revealed by XRD and Raman data, barite is present in the core of fulgurite. The presence of barite was clearly demonstrated also in the SEM analyses (Figure 14). It is interesting to note that, while quartz and feldspars were mostly observed as coarse grains in both compartments of the fulgurite, Fe oxides form the matrix between the grains. The fine-grained, crystalline matrix consists of 75–80% Fe oxide, and 20–25% of minor quartz, feldspar, and barite minerals. Grain–matrix ratios of different fulgurite sections are between 3.1 and 11.3 (mean 7.11). Similarly, SEM-BSE images and elemental mapping results (Figure 14) also confirm the mineral contents obtained in petrographic, XRD, and Raman/IR analyses. Elemental mapping of the fulgurite matrix revealed that the matrix consists of 75–85% Fe oxides.

### 3.5. Comparison of the Mineralogical Composition of Core and Shell Compartments

The results presented in Section 3.1, Section 3.2, Section 3.3 and Section 3.4 highlight the differences in the mineralogical composition of the core and shell regions of the studied fulgurite. These differences can be explained by taking into account the effects of both the diffusion of the melted material to the periphery of the fulgurite following the lightning and faster cooling at the external shell region, together with the differential properties of the various minerals (density, temperature of crystallization, polymorphism, stability).

The heavier materials diffuse slower, resulting in an increased concentration of the iron and barium-containing minerals, hematite and barite, in the core of the fulgurite. The first mineral is known to be sensitive to moisture and its relative amount diminishes in the shell of the fulgurite due to its partial transformation into goethite by meteoric water (from 8 to 4%; see Table 1) [44]. The amount of estimated goethite in the shell is 5%. However, goethite has only one iron atom in the mineral formula unit, while hematite has two, and then, the total amount of iron is in fact smaller in the shell than in the core of the fulgurite by approximately 10%, i.e., the original amount of hematite in the shell compartment of the fulgurite was, as expected, slightly smaller than in the core (by approximately 1.5%). On the other hand, near to the surface of the fulgurite, in the shell, barite could not be stabilized due to the sulfate lost (in the form of SO_2_ plus O_2_ gaseous products) [45], justifying its absence in this compartment.

The results also show that the faster cooling at the surface of the fulgurite kinetically trapped orthoclase in the shell area, while the slower cooling in the core compartment allowed for the formation of microcline, which is a low-temperature polymorph of orthoclase. This resulted in the prevalence of microcline in the core and the observation of a mixture of the two polymorphs in the shell, with the total percentage of K-feldspars decreasing only slightly from 22 to 20%, in going from the core of the fulgurite to the shell. On the other hand, the plagioclase feldspar albite and quartz exist in somewhat larger amounts in the shell compartment.

### 3.6. Fulgurite General Classification

The mineralogical composition of fulgurites is directly related to the primary material that composes them, and in fact, this is the most important argument in determining the classification criteria of fulgurites. Fulgurites can be composed of quartz-rich sands (Type 1), clayey soils (Type 2), carbonate–caliche soils (Type 3), and rocks (Type 4) [3]. Some fulgurites are called droplets and are classified as Type 5 fulgurites [3]. The studied fulgurite is similar to Type 1 fulgurites in terms of its formation. Type 1 fulgurites occur in quartz sand and often contain thin, glassy walls surrounding a hollow interior cavity. Type 1 fulgurites may contain 80% quartz and or 90% SiO_2_ in Fe–Al-rich melts [5,46,47]. The sands of the Gobi Desert are rich in quartz, K-feldspar, plagioclase, and Fe oxide minerals. In the desert environment, the effect of physical–mechanical weathering is greater than chemical weathering, and therefore feldspars do not completely decompose. Similarly, opaque minerals fragmented from the parent rock turn into Fe oxide/hydroxide minerals. As a result, the studied fulgurite belongs to the Type 1 class, and can be classified as iron-rich Type 1 fulgurite, due to its substantial contents in Fe oxide minerals.

## 4. Conclusions

In this study, a rare, iron-rich, Type 1 fulgurite sample from the Gobi Desert in Mongolia has been investigated. Too our knowledge, this is the first study on fulgurite from this geographic location reported hitherto. 

The mineral contents of the core and shell regions of the fulgurite were investigated by means of both Raman micro-spectroscopy complemented with chemometrics (principal component analysis and multivariate curve resolution methods), infrared spectroscopy, X-ray powder diffraction analysis, SEM-EBS, SEM-EDX, and polarized optical microscopy. Chemometric analysis performed on the Raman spectra allowed for a fast, non-destructive identification of the minerals, which was then confirmed by infrared spectroscopy and XRD powder analysis. The conjugation of the performed Raman spectroscopy experiments with the chemometric analysis (in particular the MCR analysis) was shown to allow for the fast identification of the minerals present in the two compartments (shell and core) of the sample, allowing for the XRD experiments to be done knowing in advance the minerals present in the samples and, this way, strongly facilitating the data analysis, which for compositionally complex samples, such as those studied in the present investigation, would have been very much challenging, if possible. XRD analysis allowed us to obtain semi-quantitative estimations of the mineral amounts present in each of the regions of the studied fulgurite. In the inner core, quartz, microcline, albite, hematite, and barite were found in amount percentages of 52, 22, 10, 8, and 8%, respectively, while in the shell, quartz (56%), a mixture of the K-feldspars microcline (9%) and orthoclase (11%), albite (15%), hematite (4%), and goethite (5%) were observed, results that are in qualitative agreement with the indications provided by the Raman experiments, and that are also consistent with the optical microscopy, SEM-BES, and SEM-EDX analyses. The differences in the minerals’ composition of the two compartments of the fulgurite, in particular the presence of orthoclase + microcline in the shell vs. the sole presence of microcline in the core, the observation of barite only in the core and of goethite only in the shell, and the predominance of hematite in the core, were explained on the basis of the effects of both the diffusion of the melted material to the periphery of the fulgurite following the lightning and the faster cooling at the external shell region, together with differential properties of the various minerals (density, temperature of crystallization, polymorphism, stability).

## Figures and Tables

**Table 1:** Properties, percentage amounts, and weight percentages of the different minerals in the core and shell compartments of the studied fulgurite, as estimated from the XRD experiments.

Mineral	Chemical Formula	Molecular Weight (g mol^−1^)	Density	Crystallization Temperature (°C)	Core		Shell	
					% (Weight)	% (Amount)	% (Weight)	% (Amount)
Quartz	SiO_2_	60.08	2.65	573 *^a^*	21	52	24	56
Microcline	KAlSi_3_O_8_	278.33	2.56	*^b^*	39	22	17	9
Orthoclase	KAlSi_3_O_8_	278.33	2.55	1170	-	-	21	11
*Total* *K-feldspars*					*39*	*22*	*38*	*20*
Albite	NaAlSi_3_O_8_	263.02	2.62	1100	19	10	30	15
Hematite	Fe_2_O_3_	159.69	5.27	2849	8	8	5	4
Goethite	FeO(OH)	88.85	4.37	*^c^*	-	-	3	5
Barite	BaSO_4_	233.39	4.48	1580	13	8	-	-
Total					100	100	100	100

*^a^* Low quartz; high quartz crystallizes at a temperature above 573 °C, but it converts spontaneously to low quartz at this temperature; *^b^* Stable below 500 °C, being a low-temperature polymorph of orthoclase. *^c^* Dehydrates to hematite at ~300 °C.

## Supplementary Materials

The following supporting information can be downloaded at: https://www.mdpi.com/article/10.3390/molecules27103053/s1, Figures S1–S7, with the assignment of the Raman spectra of the minerals observed in the studied fulgurite.

## Sample Availability

Samples of the compounds are not available.
