# Supplementary material for: Micro-Raman Spectroscopy and X-ray Diffraction Analyses of the Core and Shell Compartments of an Iron-Rich Fulgurite
Source: Molecules. 2022 May 10;27(10):3053. doi: 10.3390/molecules27103053 (PMC9144990; doi:10.3390/molecules27103053)
Supplement: Supplementary file 1 [file molecules-27-03053-s001.zip › molecules-1700635-supplementary.pdf]

Supporting Information for

Micro-Raman Spectroscopy and X-ray Diffraction Analyses of the Core and Shell Compartments of an Iron-Rich Fulgurite

Ahmet Karadag,[a] Ersin Kaygisiz [c] Timur Nikitin,[b] Sinan Ongen, [c] Gulce Ogruc Ildiz,[a,b,*]
Namık Aysal,[c] Ayberk Yilmaz[d] and Rui Fausto[b]

[a] *Department of Physics, Faculty of Sciences and Letters, Istanbul Kultur University, Istanbul, Turkey.*
[b] *University of Coimbra, CQC-IMS, Department of Chemistry, Coimbra, Portugal.*
[c] *Department of Geological Engineering, Faculty of Engineering, Istanbul University, Istanbul, Turkey.*
[d] *Department of Physics, Faculty of Science, Istanbul University, Istanbul, Turkey.*

Index

Page

Figure S1. Assignment of the Raman spectrum of quartz (α-variety) 2

Figure S2. Assignment of the Raman spectrum of albite 2

Figure S3. Assignment of the Raman spectrum of microcline 3

Figure S4. Assignment of the Raman spectrum of barite 3

Figure S5. Assignment of the Raman spectrum of hematite 4

Figure S6. Assignment of the Raman spectrum of orthoclase 4

Figure S7. Assignment of the Raman spectrum goethite 5

Assignments were based on refs. 23 to 35 in the article

***Corresponding author e-mail: g.ogruc@iku.edu.tr**

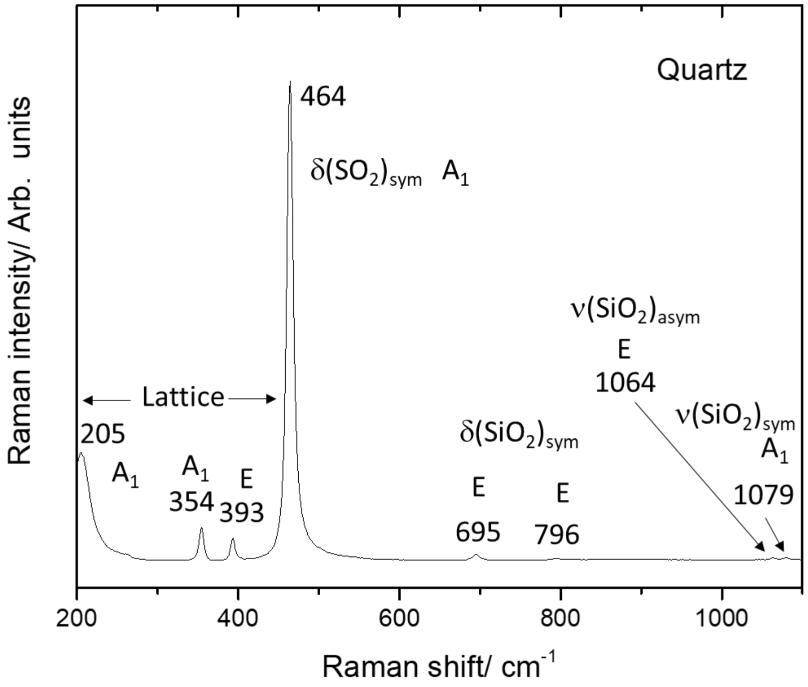

Figure S1. Assignment of the Raman spectrum of quartz (α-variety).

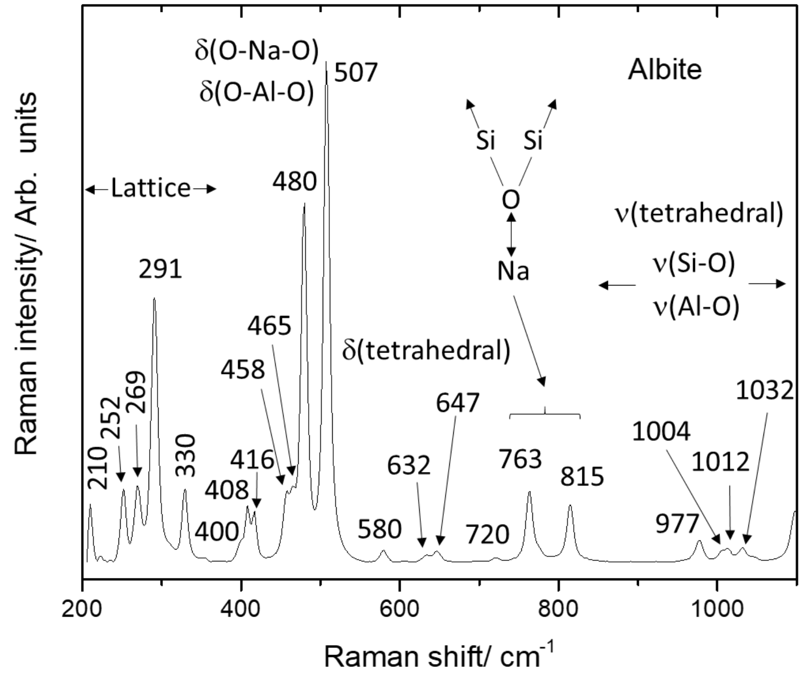

Figure S2. Assignment of the Raman spectrum of albite.

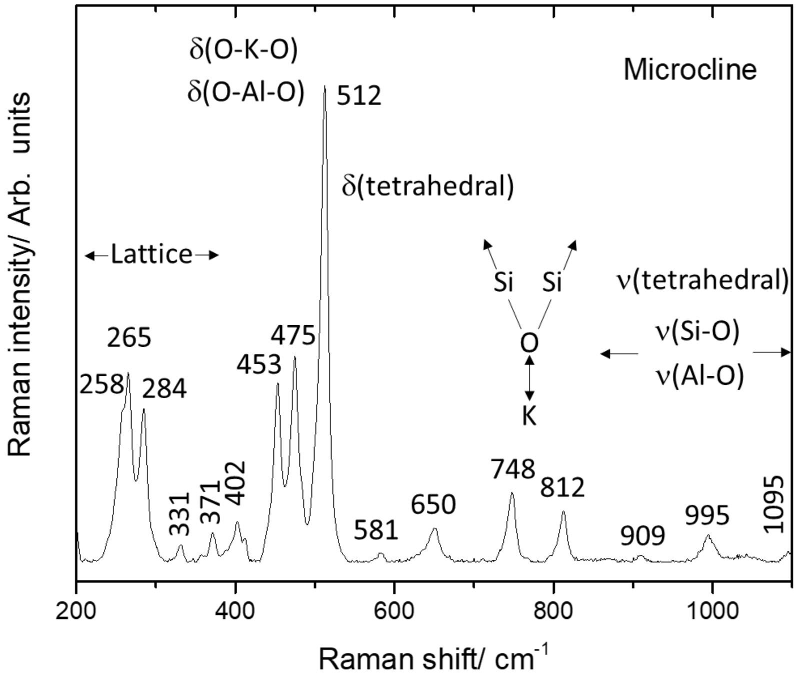

Figure S3. Assignment of the Raman spectrum of microcline.

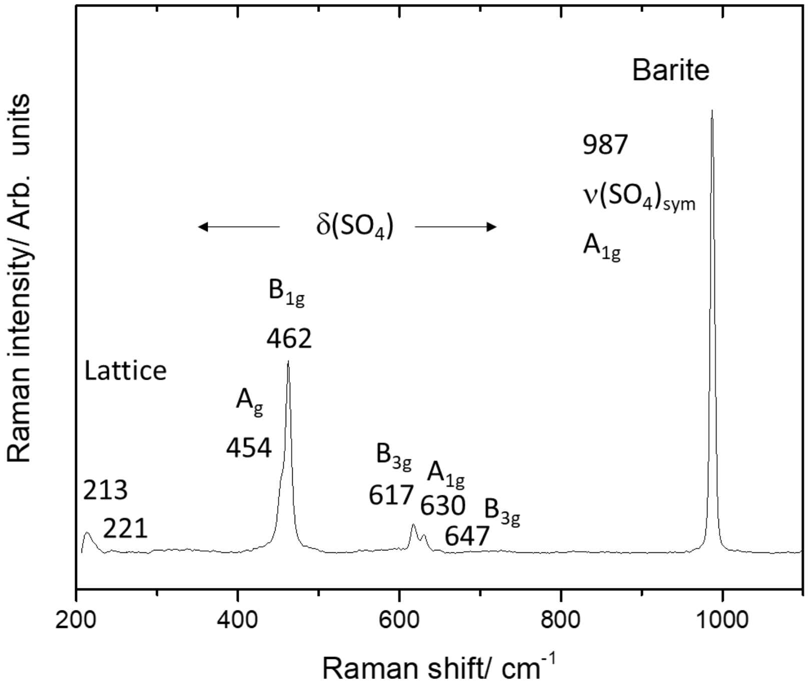

Figure S4. Assignment of the Raman spectrum of barite.

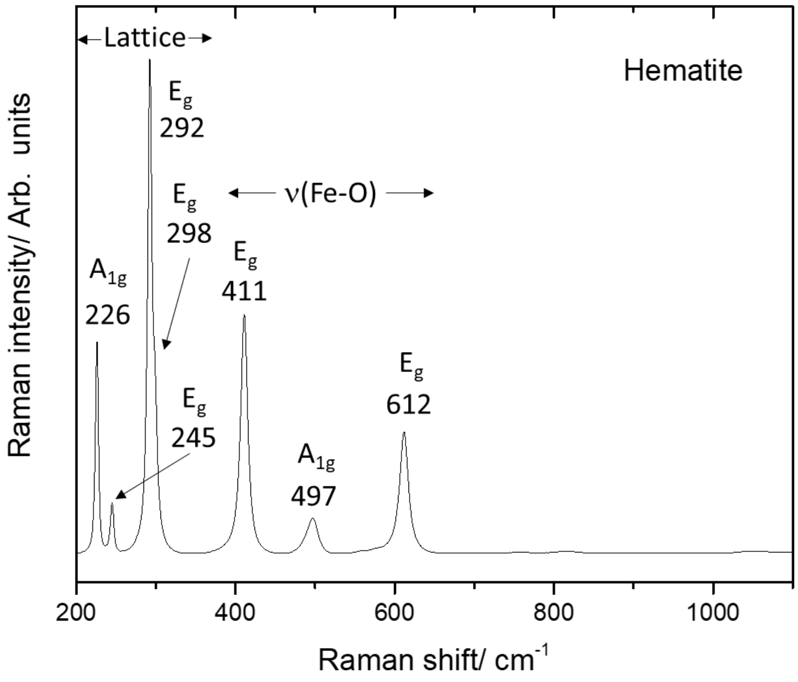

Figure S5. Assignment of the Raman spectrum of hematite.

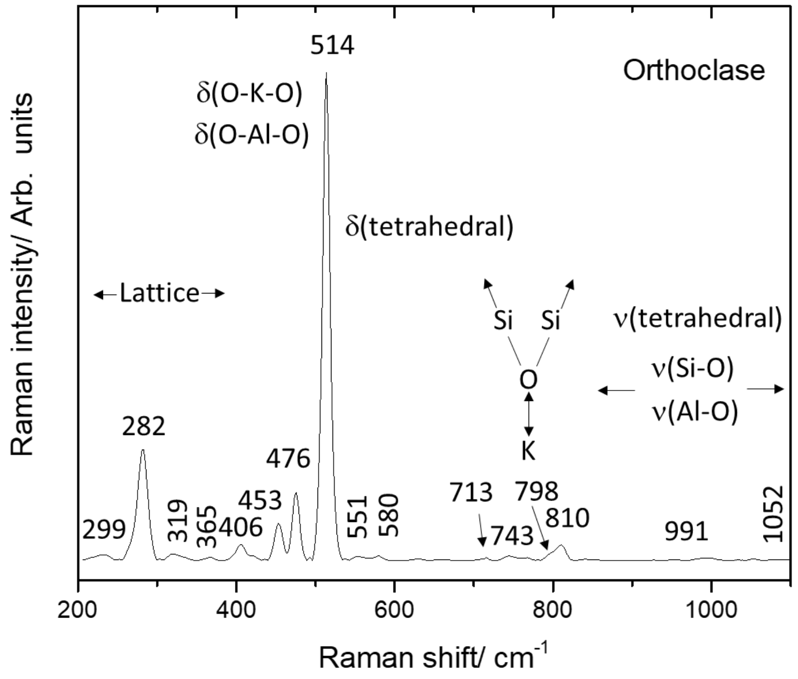

Figure S6. Assignment of the Raman spectrum of orthoclase.

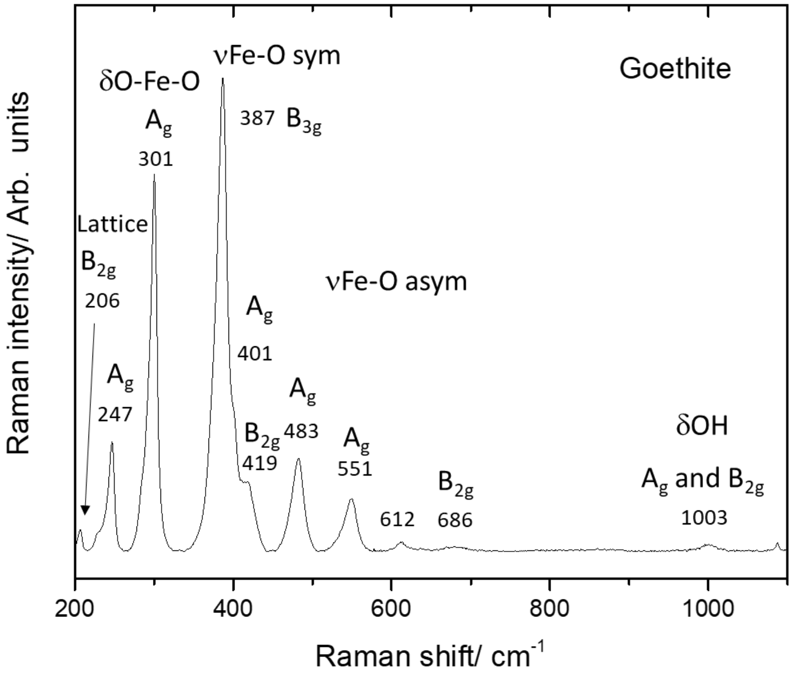

Figure S7. Assignment of the Raman spectrum of goethite.
